# Exclusive breastfeeding and mothers’ employment status in Gondar town, Northwest Ethiopia: a comparative cross-sectional study

**DOI:** 10.1186/s13006-017-0118-9

**Published:** 2017-06-17

**Authors:** Dawit Alemayehu Chekol, Gashaw Andargie Biks, Yalemzewod Assefa Gelaw, Yayehirad Alemu Melsew

**Affiliations:** 1 Ethiopian Public Health Institute, Addis Ababa, Ethiopia; 2 Department of Health Service management and Health Economics, Institute of Public Health, College of Medicine and Health Science, University of Gondar, Gondar, Ethiopia; 3 Department of Epidemiology and Biostatistics, Institute of Public Health, College of Medicine and Health Science, University of Gondar, Gondar, Ethiopia

**Keywords:** Exclusive breastfeeding, employment status, Ethiopia

## Abstract

**Background:**

Exclusive breastfeeding is defined as feeding an infant breast milk only, for the first six months. In Ethiopia, more than half of infants do not receive exclusive breastfeeding. Workplace barriers contribute to these low rates of exclusive breastfeeding practices. Understanding the sociodemographic, health related, behavioral and economic factors is crucial to promote the practice of exclusive breastfeeding in Ethiopia. Therefore, the aim of this study was to assess the extent of exclusive breastfeeding practice and associated factors among employed and unemployed mothers with children of age 7–12 months in Gondar town, northwest Ethiopia, 2015.

**Methods:**

A community based comparative cross-sectional study was conducted in October 2015. Simple random sampling technique was used to select 649 eligible mothers with children age 7–12 months during the study period. A structured and pretested interviewer administered questionnaire was used to collect the data. Three logistic regression models: whole sample, employed and not employed, were fitted.

**Results:**

A total of 649 (333 unemployed and 316 employed) mothers were interviewed. The mean duration of mothers to exclusively breastfeed was 4.77 months (± 1.36 Standard Deviation [SD]). Exclusive breastfeeding was higher among unemployed 48.0% with 95% Confidence Interval (CI) (42.0%, 54.0%) than employed (20.9%) with 95% CI (16.0%, 25.0%). Parity of three children and above (Adjusted Odds Ratio [AOR] = 3.48), and having social support (AOR = 3.45) were positively associated with exclusive breastfeeding while poor knowledge (AOR = 0.30), wealth index of the medium level (AOR = 0.38) were negatively associated among employed mothers. In the case of unemployed mothers, vaginal delivery (AOR = 2.60) and having social support (AOR = 3.03) were positively associated with exclusive breastfeeding while, poor knowledge (AOR = 0.28), and not having antenatal care (AOR = 0.56) were negatively associated.

**Conclusions:**

The overall exclusive breastfeeding practice of mothers was low. However, unemployed mothers breastfeed more than employed mothers. Providing a special support for employed mothers and revising either the legislation of the two month postpartum maternity leave or applying different alternatives is recommended.

**Electronic supplementary material:**

The online version of this article (doi:10.1186/s13006-017-0118-9) contains supplementary material, which is available to authorized users.

## Background

The experience of mothers in many countries including Ethiopia doesn’t seem to follow the expected international recommendation for exclusive breastfeeding [1]. Many infants are neither breastfed during their first hours of life with colostrum or are exclusively breastfed during their first six months. These practices may expose them to infectious diseases and have a negative impact on their growth and development [2].

Globally, the rate of exclusive breastfeeding (EBF) is 43% in 2015, while in Sub-Saharan Africa and East Africa it was 31% and 42%, respectively [3]. The prevalence of exclusive breastfeeding in Ethiopia among children of age 4-6 month was less than 50% [4–6]. In Ethiopia, the maternity leave offered during the postpartum period is only two months. This could affect working mothers not to exclusively breastfeed for the first six months [4]. Studies indicate that significant difference (10–30%) was observed between employed and unemployed mothers on the practice of exclusive breastfeeding [4, 5]. In Southeast Ethiopia, a study found that only 33% of employed mothers practiced EBF while 73% of unemployed mothers feed their children exclusively for the first six months [7]. This difference was a little lower in a study conducted in the north western part of the country which reported 44% and 65% of EBF among employed and unemployed mothers, respectively [8].

Maternal employment affects child caring time and is reported to be the major reason for low rates of EBF and also the lower duration of breastfeeding [9, 10].

The government of Ethiopia has recognized the problem of low exclusive breastfeeding practice in the country and has declared the annual “exclusive breastfeeding day” at national level, which is on 1st February [11]. Contrariwise, as the government is promoting women’s employment, with affirmative actions too, the practice of exclusive breastfeeding became lower. By investigating the level of exclusive breastfeeding practice and its associated factors in comparison with women’s employment status, this study aimed at providing the evidence for policy makers to plan and implement an approach for solving the problem.

## Methods

### Study design and setting

A community based comparative cross-sectional study was conducted from October 1-30, 2015. The two comparison study groups were employed and unemployed mothers. The study was conducted in Gondar Town, Northwest Ethiopia. Gondar town is located at 737 km from the capital of Ethiopia, Addis Ababa. The town administration is organized into 12 urban kebeles (smallest administrative units). Based on the 2007 national population census projections, the town has a total of urban population of 206,987 and among these 8002 were infants, aged less than one year, from which are 6263 were children aged between 7 and 12 months [11].

### Study population and sampling

All mothers having children of age 7-12 months and living in the town administration during the study period were included in the study. However, employed and unemployed mothers with children of age 7–12 months who were critically ill and unable to be interviewed were excluded.

The sample size was determined using Epi info statistical software version 7 by using ‘two population’ proportion formula. In a similar study conducted by Mequanint [12] prevalence of exclusive breastfeeding differences among employed and unemployed mothers were found to be 44% and 65%, respectively. A confidence level of 95%, a power of 90%, a design effect of 2% and a 5% non-response rate, the final sample size *n* = 618 + 31 = 649. Finally, 316 employed and 333 unemployed eligible mothers were taken.

**Fig. 1:**
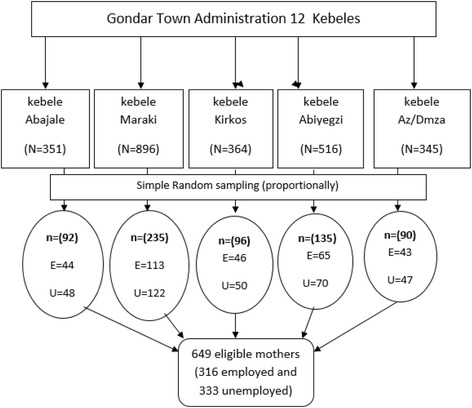
Schematic presentation of sampling procedure of the study

A multistage sampling technique was used to reach study participants. The town has 12 urban kebeles from these five kebeles were selected randomly. In the five selected kebeles, the total number of illegible mothers was estimated, household code numbers were given separately for employed and unemployed mothers. Following coding each household, the total sample size of the study was proportionally assigned to the five selected kebeles. Using the code numbers as a sampling frame study participants were selected randomly with computer generated random numbers (Fig. 1). Households were visited during working days to interview unemployed mothers whereas employed mothers were interviewed during the weekends.

### Data collection and analysis

The pretested structured interviewer administered questionnaire was used to collect data. The questionnaire consisted of six parts: sociodemographic characteristics, economic indicators, birth related factors, exclusive breastfeeding related factors, knowledge, attitude and practice about exclusive breastfeeding and health service related factors.

The questionnaire was first developed in English and translated to Amharic, the local language. Ten trained data collectors collected the data under the continuous supervision of principal investigators.

Data were entered using EPI-INFO version 3.5.3 and then exported to SPSS version 20 for analysis. Descriptive and summary statistic was carried out to describe study participants according to different characteristics, and proportions were computed to find out the prevalence of exclusive breastfeeding (EBF). Binary logistic regression was fitted to identify factors associated with exclusive breastfeeding practice. Three models were fitted; first for the whole sample (employed and unemployed mothers combined), second for employed mothers only and the third for unemployed mothers. Adjusted odds ratios (AOR) with 95% confidence interval (CI) were used to measure the strength and significance of the association. The *p* values of less than 0.05 were considered as a statistically significant association.

### Terms and operational definitions

Employed mothers: mothers who work outside the home for income in addition to the work they perform at home in raising their children [13].

Knowledge of mother: mothers’ information on the advantages and recommended duration of exclusive breastfeeding. Mothers were asked six knowledge related questions and each correct answer was given a value of 1 and an incorrect answer a value of 0. After computing the sum for each respondent and mean, it was dichotomized into good knowledge ≥ mean, poor knowledge < mean.

Attitude: there were three attitude related questions and mothers who respond the right response to all the three questions the will be categorized as having a good attitude.

Practiced EBF: mothers who fed breast milk only for the first six months.

Predominant breastfeeding: the practice of feeding breast milk along with water [1].

Mixed breastfeeding: the practice of feeding breast milk along with food [1].

Early initiation of breastfeeding: infants who were put to the breast within one hour of birth [1].

Prelacteal feeding: children who have been given something other than breast milk during the first three days of life [1].

## Results

### Sociodemographic characteristics

A total 649 mothers having children of age 7–12 months were interviewed. The mean age mothers were 28.4 years (± 4.8 years) and the mean age of children was 8.9 months (± 3.604 months). Of the employed mothers 96 (30.4%) were government employed. The majority, 89.5%, of the participants attended secondary education and above (Table 1).

**Table 1:** Sociodemographic, knowledge and attitude characteristics of employed and unemployed mothers in Gondar town, northwest Ethiopia, October 2015

Variables	Maternal employment	
	Employed *n* = 316	Unemployed *n* = 333
	Number (%)	Number (%)
Sex of child		
Male	150 (47.5)	173 (52.0)
Female	166 (52.5)	160 (48.0)
Maternal age		
18-23	25 (7.9)	59 (17.7)
24-29	153 (48.9)	155 (46.5)
> = 30	138 (43.7)	119 (35.7)
Parity		
1–2 children	176 (55.7)	215 (64.6)
3 and above children	140 (44.3)	118 (35.4)
Marital status		
Married	267 (84.5)	314 (94.2)
Single	17 (5.4)	1(0.3)
Divorced	26 (8.2)	14 (4.2)
Widowed	6 (1.9)	4 (1.2)
Ethnicity		
Amhara	243 (76.9)	279 (83.8)
Qimant	26 (8.2)	17 (5.1)
Tigrie	38 (12.0)	36 (10.8)
Others	9 (2.8)	1 (0.3)
Maternal education		
No formal education	35 (11.1)	45 (13.5)
Only read and write	33 (10.4)	59 (17.7)
Primary	36 (11.4)	81 (24.3)
Secondary	114 (36.1)	122 (36.6)
Higher	98 (31.0)	26 (7.8)
Husband education		
No formal education	21 (11.1)	36 (10.8)
Can read and write	35 (10.4)	60 (18.0)
Primary	34 (11.4)	58 (17.4)
Secondary	104 (36.1)	118 (35.4)
Higher	122 (31.0)	61 (18.3)
Religion		
Orthodox Christian	228 (72.2)	212 (63.7)
Muslim	74 (23.4)	109 (32.7)
Protestant	10 (3.2)	6 (1.8)
Catholic	3 (0.9)	2 (0.6)
Other	1 (0.3)	4 (1.2)
Attitude towards EBF		
Good attitude	252 (79.7)	287 (86.2)
Knowledge on EBF		
Good knowledge	253 (80.1)	271 (81.4)

### Knowledge and attitude of mothers towards exclusive breastfeeding

The majority, 80.0%, and 83.1%, of participants, reported they had good knowledge and attitude about exclusive breastfeeding, respectively. The proportion of knowledge on has no significance difference in employment status (Table 1).

### Health related characteristics

The majority, 89.6%, of participants (employed = 282: unemployed = 300) gave birth at health center by a skilled attendant. The majority of participants, 94.5% and 92.2%, practiced breastfeeding their children immediately within one hour of birth, respectively. Furthermore, 92.6% and 98.5%, employed and unemployed mothers were informed about exclusive breastfeeding during any of their antenatal care visits and had support from their partner, respectively (Table 2).

**Table 2:** Health service related factors of employed and unemployed mothers in Gondar town, northwest Ethiopia, October, 2015

Variables	Maternal employment	
	Employed (*n* = 316)	Unemployed (*n* = 333)
Place of birth		
Home	34 (10.8)	33 (9.9)
Health facility	282 (89.2)	300 (90.1)
Mode of delivery		
Vaginal delivery	258 (81.7)	279 (83.7)
Caesarean section	58 (18.3)	54 (16.3)
Timely initiation of BF / within 1 h		
Yes	205 (64.9)	236 (70.1)
Any non-breastmilk given during the first 3 days		
Yes	73 (23.1)	81 (24.3)
Frequency of EBF/24 h		
< 9 times	132 (41.8)	184 (58.2)
9 and above times	94 (28.2)	239 (71.8)
Having social support		
Yes	234 (74.1)	289 (86.9)
Antenatal care		
Yes	232 (73.4)	248 (74.5)

The main reasons reported by employed mothers for not practicing exclusive breastfeeding were that working was a hindrance 84 (26.6%), and concern about the amount of milk 23 (7.3%) immediately after birth.

### Exclusive breastfeeding

The overall exclusive breastfeeding practice was 34.8% (95% CI 33.0%, 36.7%). The mean (SD) duration that infants received breast milk only was 4.8 (± 1.4) month. A total of 20.9% (95% CI 16.0%, 25.0%) employed mothers and 48.0% (95% CI 42.0%, 54.0%) unemployed mothers practiced exclusive breastfeeding.

### Factors associated with exclusive breastfeeding practice

The descriptive statistics comparing sociodemographic, knowledge and attitude characteristics showed a significant difference along the employment status of mothers. Exclusive breastfeeding, marital status, attitude, social support and household socioeconomic positions were significantly different along with the employment status of mothers (Additional file 1).

We fitted three different models to assess exclusive breastfeeding practice. The first model was fitted to assess the overall factors of exclusive breastfeeding practice. Variables such as knowledge, attitude, and employment status were significantly associated with exclusive breastfeeding practice regardless of employment status.

Mothers who had poor attitude (AOR 0.13, 95% CI 0.05, 0.31), and poor knowledge (AOR 0.44, 95% CI 0.24, 0.78) were less likely to practice exclusive breastfeeding than their counterparts. Mothers who were unemployed were 3.4 times more likely to practice exclusive breastfeeding than employed mothers (AOR 3.43, 95% CI 2.38, 4.95) (Table 3).

**Table 3:** Independent predictors of exclusive breastfeeding for both employed and unemployed mothers (Full model) in Gondar town, northwest Ethiopia, October, 2015

Variables	Exclusive breastfeeding		COR (95% CI)	AOR (95% C.I)	*p*-value
	Yes	No			
Birth Interval					
1–2 years	131	276	0.73 (0.53,1.02)	0.72 (.50, 1.05)	0.085
3 and above	95	147	1	1	
Knowledge					
Poor knowledge	19	106	0.27(0.16, 0.46)	0.437 (0.245, 0.779)	0.005
Good knowledge	207	317	1	1	
Place of birth					
Home	12	55	0.38 (0.20, 0.72)	0.53 (0.26, 1.09)	0.084
Health center	214	368	1	1	
Marital status					
Married	215	366	3.04 (1.56, 5.93)	1.88 (0.91,3.89	0.087
Unmarried	11	57	1	1	
Employment status					
Unemployed	160	173	3.50 (2.48, 4.95)	3.43 (2.38, 4.95)	.0001
Employed	66	250	1	1	
Attitude towards EBF					
Poor Attitude	6	103	0.84 (0.04, 0.20)	0.13 (0.05, 0.31)	.0001
Good Attitude	220	319	1	1	

*EBF* exclusive breastfeeding, *COR* Crude odds ratio and *AOR* adjusted odds ratio

The second model was fitted only for unemployed mothers. Accordingly, knowledge on exclusive breastfeeding, social support, history of antenatal (ANC) care follow up and mode of delivery showed a significant association (Table 4). Unemployed mothers who had poor knowledge on exclusive breastfeeding were 72.2% less likely to practice exclusive breastfeeding compared to those who had good knowledge (AOR 0.28, 95% CI 0.14, 0.54) and mothers who had no social support were 66.6% less likely to breastfeed exclusively as compared with those who were supported (AOR 0.33, 95% CI 0.16, 0.71). In addition, mothers who had antenatal care follow up as per the recommended frequency were 1.8 times more likely to breastfeed exclusively as compared with to those who had no antenatal care follow up (AOR 0.56, 95% CI 0.32, 0.98) and mothers who birthed by spontaneous vaginal mode were 2.5 times more likely to breastfeed exclusively when compared with those who were delivered by caesarean section (AOR 2.60, 95% CI 1.33, 5.08).

**Table 4:** Multivariate analysis of exclusive breastfeeding among unemployed mothers Gondar town, northwest, October, 2015

Variables	Exclusive breastfeeding		COR (95% CI)	AOR (95% CI)	*p* - value
	Yes	No			
Birth Interval					
1–2 years	92	120	0.60 (0.38, 0.94)	0.63 (0.38, 1.03)	0.085
3 and above	68	53	1	1	
Knowledge on EBF					
Poor knowledge	14	48	0.250 (0.13, 0.47)	0.28 (0.14, 0.54)	0.0001
Good knowledge	146	125	1	1	
Social support					
No	11	33	0.31 (0.15, 0.64)	0.33 (0.16, 0 .71)	0.004
Yes	149	140	1	1	
Place of birth					
Home	9	24	0.37 (0.17, 0.82)	0.47 (0.20, 1.10)	0.082
Health center	151	149	1	1	
Antenatal care					
No	29	56	0.46 (0.28, 0.77)	0.56 (0.32, 0.98)	0.042
Yes	131	117	1	1	
Mode of delivery					
Vaginal	145	134	2.81 (1.48, 5.34)	2.60 (1.33, 5.08)	0.005
CS	15	39	1	1	

*EBF* exclusive breastfeeding, *COR* Crude odds ratio and *AOR* adjusted odds ratio

The third model was fitted for employed mothers. As a result, employed mothers who had three and above children were 3.5 times more likely to breastfeed exclusively than those who had below three children (AOR 3.48, 95% CI 1.79, 6.78), employed mothers who had good knowledge of exclusive breastfeeding were more likely exclusively breastfeed (AOR 0.30, 95% CI 0.11, 0.80), and also mothers who had no social support were 3.5 times more likely to breastfeed exclusively than those who have social support (AOR 3.46, 95% CI 1.78, 6.72) (Table 5).

**Table 5:** Multivariate analysis of exclusive breastfeeding among employed mothers in Gondar town, northwest Ethiopia, October, 2015

Variables	Exclusive breastfeeding		COR (95% CI)	AOR (95% CI)	*p* - value
	Yes	No			
Parity					
1–2 children	130	46	1	1	0.085
3 and above	120	20	2.12 (1.19, 3.79)	3.48 (1.79, 6.78)	0.0001
Knowledge on BF					
Good knowledge	61	192	1	1	
Poor knowledge	5	58	0.27 (0.10, 0.71)	0.30 (0.11, 0.80)	0.016
Social support					
Yes	58	26	1	1	
No	194	40	2.17 (1.26, 4.01)	3.46 (1.78, 6.72)	0.0001
Place of birth					
Home	31	3	1	1	
Health center	219	63	0.34 (0.10, 1.14)	0.36 (0 .10, 1.28)	0.115
Wealth					
Lower	13	71	1	1	
Medium	24	85	0.593 (0.288,1.223	0.38 (0.17, 0.85)	0.018*
Higher	29	94	0.915 (0.495, 1.693)	1.00 (0.52, 1.93)	0.997

*EBF* exclusive breastfeeding, *COR* Crude odds ratio and *AOR* adjusted odds ratio

Likewise, women in the wealth index ranking middle were 62% less likely to EBF than lower rank (AOR 0.38, 95% CI 0.17, 0.85).

## Discussion

As a global public health recommendation, infants should be fed breast milk only for the first six months, because breastmilk is hygienic compared with other fluids and contains all the nutrients and antibodies that are very important to prevent disease months [14]. However, employed mothers may return to work early after giving birth for various reasons. If not supported by their employers, they can be separated from their babies, have difficulty expressing and storing milk and thus not be able to maintain exclusive breastfeeding [10].

This study revealed that the prevalence of exclusive breastfeeding practice was 20.9% among employed mothers and 48.0% among unemployed mothers. This result was lower for both employed and unemployed mothers as compared to the study conducted in different parts of Ethiopia such as; a study conducted in Mecha found 44% of employed and 65% unemployed mothers practice [12], and another study found 33% of employed and 73% of unemployed mothers breastfed their children for six months exclusively [7].

Employment status was significantly associated with exclusive breastfeeding practice which was supported in a study conducted in Goba, southern Ethiopia [7]. In this study, unemployed mothers were more likely to practice EBF than employed ones. This finding was consistent with studies done in Saudi Arabia [15], Canada [16] and Guatemala [17]. This might be due to the fact that unemployed mothers get a longer time to stay with their children [18]**.** Similar findings were found in many studies which confirmed that a mothers’ work status has a negative association with exclusive breastfeeding practices. In addition to EBF, the working status of mothers also shorten the duration of breastfeeding [19, 20]. A study in Guatemala city also confirmed that women who did not work outside the home were 32% times more likely to exclusively breastfeed as were women who worked outside the home [17].

The majority of employed mothers started breastfeeding their children with liquids and food supplementations earlier as compared to their unemployed counterparts and they frequently attribute early weaning to unsupportive work environments [21]. The possible reason might be employment rules and regulations, such as less maternity leave (three months in Ethiopian context), and employed mothers have less opportunity to stay at home, compromising exclusive breastfeeding and lack child care facilities close to the workplace [20].

In this study mothers who have three and above children were 3.5 times more likely to breastfeed exclusively than those who had one or two children. This could be because of the fact that multipara mothers might be more experienced and knowledgeable on the advantage of exclusive breastfeeding [22]. This finding is inconsistent with studies done in Saudi Arabia and Canada [16, 23]. In contrast, a study done in Bahirdar, Ethiopia reported that mothers who were primipara were two times more likely to exclusively breastfeed than multipara mothers [8].

According to our study, mothers who had poor knowledge about the recommended duration of EBF were 70.4% less likely to breastfeed exclusively than those who had good knowledge, which is similar to results found in Southern Ethiopia [24].

Employed mothers ranking a middle wealth index scores were associated with shorter duration of exclusive breastfeeding than the lower ranking. The plausible reason might be that middle wealthy mothers were in good economic conditions, had less stress conditions and could use other breastmilk substitutes [18]. But this finding contradicts with results found in a study conducted in Ethiopia in that being middle/ richer/richest wealth index was retained as positive predictors of EBF [5] and also a study was done in Sudan [25].

Regarding the associated factors of exclusive breastfeeding among unemployed mothers, those who had poor knowledge on exclusive breastfeeding were less likely to breastfeed exclusively than those who had good knowledge. This result is supported by Mecha and southern Ethiopia findings. This might be due to unemployed mothers having poor knowledge did not understand the disadvantages of EBF [22].

Social support is another factor important for breastfeeding [20]. However, in this study employed mothers who were not socially supported were more likely to breastfeed exclusively than those who have social support. The possible explanation for this might be these mothers were obliged to leave their children with somebody else who care for their children at home. That means mothers who want to leave their children for work may search and find someone to take care of their children. Thus, these mothers present with higher social support during the survey.

Unlike their employed counterparts, in this study, unemployed mothers who had no social support were less likely to breastfeed exclusively than those who were supported. A similar finding in Goba showed support from spouse favors mothers to exercise exclusive breastfeeding [7]. In the same way results in the USA showed that a positive influence of support for breastfeeding initiation and duration in that 57% of mothers considered their support group to be lightly or moderately important in influencing their decision to breastfeed beyond a year [26]. The possible explanation could be mothers who had a social support have the benefit of improving satisfaction with the infant feeding experience with people around them [27].

Unemployed mothers who had no ANC follow up as per the recommended frequency were 43.7% less likely to breastfeed exclusively than those who had ANC follow up. This was supported by findings in America where black mothers who had an antenatal care breastfed their infants for longer durations [28]. The possible reason might be antenatal care based educational programs had the greatest effect of any single intervention on both initiation and duration of EBF [29].

Those unemployed mothers who delivered vaginally were more likely to breastfeed exclusively than those who were delivered by caesarean section. This finding was consistent with a study from Canada [16]. The rationale behind might be due to the fact that mothers who delivered by caesarean section often find it difficult to achieve a comfortable position for breastfeeding.

### Limitations of the study

This study was not supplemented with a qualitative study. We didn’t study the employment characteristics, such as time and sector. There might be misclassification of mothers on their attitude status due to the fewer number of questions. There might be recall bias since mothers were interviewed to recall their experience. Our data collection tool was not validated this might limit the findings generalizability.

## Conclusion

A large proportion of infants were not exclusively breastfed by both employed and unemployed mothers. The duration of EBF was below the WHO recommendation and the target of the Ethiopian health sector development plan. This study has indicated employed mothers are less likely to practice exclusive breastfeeding than unemployed mothers. The exclusive breastfeeding status of unemployed mothers was significantly better than that of employed mothers. Parity, knowledge about exclusive breastfeeding, social support, and maternal wealth index were independent predictors of exclusive breastfeeding among employed mothers. While the knowledge on exclusive breastfeeding, social support, antenatal care and mode of delivery were independent predictors of exclusive breastfeeding of unemployed mothers. A different approach of intervention should be implemented to employed and unemployed mothers to promote exclusive breastfeeding.

## Additional file

Additional file 1:
**Table S1.** Descriptive statistics on demographic, knowledge and attitude characteristics of mothers having children of age 7–12 months in Gondar town, October 2015. (DOCX 14 kb)

## Abbreviations

ANC: antenatal care
BF: breastfeeding
CSA: Central statistical agency
EBF: exclusive breastfeeding
EBFP: exclusive breastfeeding practice
EDHS: Ethiopian demographic and health survey
FMOH: Federal ministry of health
HIV: Human immunodeficiency virus
HSDP: Health sector development program
IYCF: Infant and young child feeding
NGO: Non - Governmental organization
MDG: Millennium development goal
ORS: Oral rehydration salt
SDG: Sustainable development goals
SSSF: Solid Semi-Solid and soft food
ANRS: Amhara national regional state
UNICEF: United nations children’s fund
USAID: United State agency for international development
WHO: World health organization
