# Supplementary material for: Exclusive breastfeeding and mothers’ employment status in Gondar town, Northwest Ethiopia: a comparative cross-sectional study
Source: Int Breastfeed J. 2017 Jun 17;12:27. doi: 10.1186/s13006-017-0118-9 (PMC5473972; doi:10.1186/s13006-017-0118-9)
Supplement: Additional file 1: — Table S1. Descriptive statistics on demographic, knowledge and attitude characteristics of mothers having children of age 7–12 months in Gondar town, October 2015. (DOCX 14 kb) [file 13006_2017_118_MOESM1_ESM.docx]

Table S1. Descriptive statistics on demographic, knowledge and attitude characteristics of mothers having children of age 7-12 months in Gondar town, October 2015

| **Variable** | **Employment status** | | **P - value** |
| --- | --- | --- | --- |
|  | **Unemployed** | **Employed** |  |
| **Exclusive BF** |  |  | **<0.001** |
| No | 173(26.7%) | 250(38.5%) |  |
| Yes | 160(24.7%) | 66(10.2%) |  |
| **Birth Interval** |  |  | 0.607 |
| 1-2 | 212 (32.7%) | 195 (30.0%) |  |
| 3 and above | 121 (18.6%) | 121 (18.6%) |  |
| **Knowledge** |  |  | 0.67 |
| Poor knowledge | 62 (9.6%) | 63(9.7%) |  |
| Good knowledge | 271 (41.8%) | 253(39.0%) |  |
| **Place of delivery** |  |  | 0.722 |
| Home | 33 (5.1%) | 34 (5.2%) |  |
| Health Centre | 300 (46.2%) | 282 (43.5%) |  |
| **Marital status** |  |  | **<0.001** |
| Married | 314(48.4%) | 267(41.1%) |  |
| Unmarried | 19(2.9%) | 49(7.6%) |  |
| **Attitude** |  |  | **0.023** |
| Poor attitude | 45(6.9%) | 64(9.9%) |  |
| Good attitude | 287(44.3%) | 252(38.9%) |  |
| **Social support** |  |  | **<0.001** |
| No | 44(6.8%) | 82(12.6%) |  |
| Yes | 289(44.5%) | 234(36.1%) |  |
| **ANC** |  |  | 0.934 |
| Yes | 269(41.6%) | 256(39.6%) |  |
| No | 62(9.6%) | 60(9.3%) |  |
| **Wealth Index** |  |  | **0.01** |
| Poor | 131(20.2%) | 84(12.9%) |  |
| Medium | 109(16.8%) | 109(16.8%) |  |
| Rich | 93(14.3%) | 123(19.0%) |  |
